# Gap between the Scientificization and Utilization of Korean Medicine for Depressive Disorder in South Korea with the Highest Suicide Rate among OECD Countries

**DOI:** 10.3390/jcm11237022

**Published:** 2022-11-28

**Authors:** Chan-Young Kwon

**Affiliations:** Department of Oriental Neuropsychiatry, Dong-Eui University College of Korean Medicine, 52-57 Yangjeong-ro, Busanjin-gu, Busan 47227, Republic of Korea; beanalogue@deu.ac.kr; Tel.: +82-51-850-8808

**Keywords:** depression, depressive disorders, suicide, Korea, integrative medicine, Korean medicine

## Abstract

In South Korea, with the highest suicide rate among Organization for Economic Co-operation and Development countries, doctors of both Korean medicine (KM) and Western medicine (WM) are licensed in the national healthcare system. The beneficial effects of complementary and integrative medicine, including KM, for depressive disorders, a major cause of suicide, have been reported in some clinical studies. Longitudinal change (2012–2021) of KM and WM use for depressive disorders and the attempts to scientificize KM in the context of depressive disorders were investigated. Specifically, clinical practice guidelines (CPGs) and national R&D projects of KM in the treatment of depressive disorders were investigated. The use of KM treatment for patients with a depressive disorder appears to be gradually decreasing in South Korea (annual number of patients in 2012 and 2021: 3607 and 2151 (−40.37%)), while the use of WM treatment is increasing (662,947 and 989,909 (+49.32%)). With the support of the government, KM CPG for depressive disorders and some R&D projects on KM for depressive disorders are being implemented. Our findings highlight the gap between the accumulation of clinical evidence, or the government’s efforts to promote the evidence-based use of KM for depressive disorders, and its practical use in South Korea.

## 1. Introduction

Globally, depressive disorders are the psychiatric disorder that causes the greatest disease burden [1,2], and the coronavirus pandemic of 2019 (COVID-19) has been considered an aggravating factor that has intensified the burden [3]. Although the socioeconomic burden caused by depressive disorders has been increasing, it was found that there are still substantial unmet treatment needs [2]. Suicide, the most catastrophic result of depressive disorders, is increasing worldwide and accounted for 1.4% of premature deaths worldwide in 2015 [4]. Moreover, it is estimated that the number of suicide attempts is about 30 times the suicide cases [4]. South Korea has the highest suicide rate among the Organization for Economic Co-operation and Development (OECD) countries [5], and the increasing prevalence of depression (i.e., 5.3% in 2013) has been identified as one of the leading causes of the high suicide rate in South Korea [6].

Conventional medicine or Western medicine (WM) approaches to treat depressive disorders from the viewpoint of evidence-based medicine (EBM) include antidepressants, especially second-generation antidepressants. As a non-pharmacological treatment, cognitive behavioral therapy is also considered to have reliable supporting evidence [7]. A recent network meta-analysis of 101 randomized controlled trials found that a combination of pharmacotherapy and psychotherapy was the best therapeutic option for individuals with moderate depression [8]. However, in the context of suicide, the therapeutic risk management of the suicidal patient should be introduced in a multidisciplinary fashion [9]. Additionally, the effect of antidepressants on suicide risk in WM is controversial [10], and some favorable results have been reported for ketamine [11] or neuromodulation, including electroconvulsive therapy [12], but the evidence is still considered provisional. Therefore, a comprehensive management strategy is needed to manage suicide risk rather than just one or two interventions. In some countries, complementary and integrative medicine (CIM) modalities may be considered promising options for constructing the multidisciplinary strategy.

Although the level of evidence for most CIM modalities for depression is considered weak [13,14], recent clinical evidence has supported the claim that acupuncture is effective in the treatment of patients with a major depressive disorder when combined with WM approaches such as serotonin-norepinephrine reuptake inhibitors [8]. Additionally, the prevalence of CIM use in the treatment of psychiatric disorders, including depression, is increasing, because of the limitations of conventional medicine for depression [15,16,17]. In some Asian countries, including South Korea, licensed clinicians prescribe CIM modalities based on established medical systems, such as traditional Chinese medicine and Korean medicine (KM) [18]. KM doctors provide CIM modalities to patients from the perspective of EBM, and the Korean government has been promoting the development of evidence-based KM clinical practice guidelines (CPGs) since 2016 to help scientificize and disseminate KM services [19].

In view of this, it seems reasonable to establish a national suicide-prevention strategy that utilizes CIM, including KM, in South Korea, which consistently ranks first in suicide rate among OECD countries [5]. In addition, the ongoing evidence-based KM CPG of depression (to be published in February 2024) may be considered an important data source in suicide-prevention strategies where multidisciplinary management is important [9]. However, there have been no studies examining the applicability of CIM in the context of suicide prevention or management of depressive disorders in South Korea. Therefore, in this study, the author attempted to comparatively analyze the trends in the use of KM and WM for the treatment of depressive disorders by analyzing the national health insurance system of South Korea. Moreover, the author summarized the attempts to scientificize KM in the context of depressive disorders, and discussed the gap between the scientificization and utilization of KM for depressive disorders in South Korea. Ultimately, the purpose of this communication is to discuss the potential use and challenges of KM in solving the high-suicide-rate issue in South Korea in the context of multidisciplinary management.

## 2. Materials and Methods

### 2.1. Trend Analysis of KM and WM Use for Depressive Disorders

#### 2.1.1. Database

The Healthcare Bigdata Hub (https://opendata.hira.or.kr/ accessed on 17 July 2022), an open system provided by the Korean Ministry of Health and Welfare, has provided statistics since 2010 for all diseases treated under the health insurance system, which has been compulsory for over 99% of Koreans [20]. This database can be accessed by anyone to identify the data without request or permission to use the data, and does not contain any patient identification information. The researchers used this database to analyze the patterns of medical use of patients who received treatment for depressive disorders as the main diagnosis between 2012 and 2021 (10 years in total).

#### 2.1.2. Definition of Depressive Disorders

As the database uses the Korean Standard Classification of Diseases, 7th Revision (KCD-7), based on the International Classification of Diseases, 10th Revision (ICD-10) code, patients with depressive disorders were defined as having a main diagnosis of F32 (Depressive episode), F320 (Mild depressive episode), F321 (Moderate depressive episode), F322 (Severe depressive episode without psychotic symptoms), F323 (Severe depressive episode with psychotic symptoms), F328 (Other depressive episodes), F329 (Depressive episode, unspecified), F33 (Recurrent depressive disorder), F330 (Recurrent depressive disorder, current episode mild), F331 (Recurrent depressive disorder, current episode moderate), F332 (Recurrent depressive disorder, current episode severe without psychotic symptoms), F333 (Recurrent depressive disorder, current episode severe with psychotic symptoms), F334 (Recurrent depressive disorder, currently in remission), F338 (Other recurrent depressive disorders), or F339 (Recurrent depressive disorder, unspecified). Additionally, U221 (Stagnation syndrome) and U222 (Hwabyung), which are considered depressive disorder concepts in KM [21,22], were considered as additional options for depressive disorders.

#### 2.1.3. Statistical Analysis

The collected information includes number of patients (yearly), number of visit days (yearly), number of claims (yearly), government insurance total cost (yearly, KRW), and out-of-pocket total cost (yearly, KRW) in TKM and WM clinics. The data were organized and analyzed using the program Microsoft Excel (Microsoft 365, DC: Microsoft, Redmond, WA, USA). Additionally, trend changes in KM and WM use for depressive disorders in South Korea were visualized.

### 2.2. Analysis of Efforts to Scientificize KM in the Context of Depressive Disorders

#### 2.2.1. Clinical Practice Guidelines

There are two government-funded research institutes wholly related to KM in South Korea: one is the Korea Institute of Oriental Medicine (KIOM), and the other is the National Institute for Korean Medicine Development (NIKOM). On the official website of KIOM (https://www.kiom.re.kr/eng/ accessed on 15 October 2022), evidence-based KM CPGs for some clinical conditions developed by this institution are provided. Additionally, on the official website of NIKOM (https://nikom.or.kr/engnikom/index.do accessed on 15 October 2022), evidence-based KM CPGs based on the Korean government’s KM standardization efforts started in 2016 are provided. In particular, NIKOM’s website contains almost all of KM CPGs developed by other institution in KM society as well as CPGs developed by NIKOM. The authors searched the official homepages of the two institutions to collect official efforts to scientize KM in the context of depressive disorders in South Korea.

#### 2.2.2. Research and Development

In order to investigate research and development (R&D) on KM for depression supported by the Korean government, National Science and Technology Information Service (https://www.ntis.go.kr/ accessed on 15 October 2022), a database that offers R&D information in South Korea, were searched. The keywords for searching were depression and KM. The search was conducted on 15 October 2022.

## 3. Results

### 3.1. Trend of KM and WM Use for Depressive Disorders in South Korea

#### 3.1.1. Number of Patients (Yearly)

**Figure 1:**
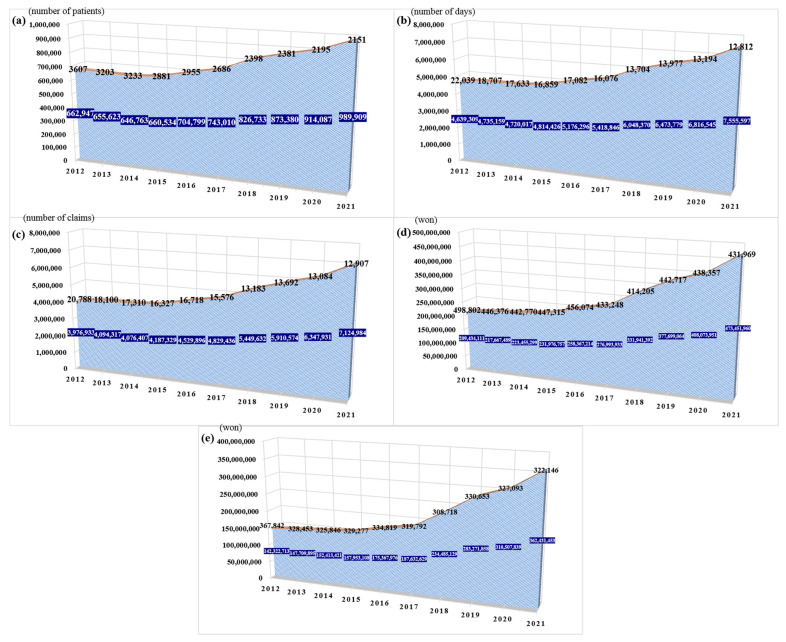
Changes in the use of Western (blue color) and Korean medicine (orange color) in patients with depressive disorders over the past 10 years in South Korea (2012–2021). (**a**) Number of patients (yearly); (**b**) Number of visit days (yearly); (**c**) Number of claims (yearly); (**d**) Government insurance total cost (yearly); and (**e**) Out-of-pocket total cost (yearly). Note. Patients with depressive disorders were defined as having a main diagnosis of F32, F320, F321, F322, F323, F328, F329, F33, F330, F331, F332, F333, F334, F338, or F339 (International Classification of Diseases code).

In 2012 (baseline), 2016, and 2021, the annual number of patients who used a WM service for depressive disorders as their main diagnosis was 662,947, 704,799 (+6.31%, compared to baseline value), and 989,909 (+49.32%, compared to baseline value), respectively, while the annual number of patients who used the KM service was 3607, 2955 (−18.08%), and 2151 (−40.37%), respectively (Figure 1a). Even when we considered stagnation syndrome (code: U221) and Hwabyung (code: U222) as depressive disorders, similar decreases were observed over the same period, at 31,539, 24,686 (−21.73%), and 21,072 (−33.19%), respectively (Table 1).

#### 3.1.2. Number of Visit Days (Yearly)

In 2012, 2016, and 2021, the annual number of visit days for WM services by patients with depressive disorders was 4,639,309, 5,176,296 (+11.57%), and 7,555,597 (+62.86%), respectively, while the annual number for KM services was 22,039, 17,082 (−22.49%), and 12,812 (−41.87%), respectively (Figure 1b). When we considered stagnation syndrome and Hwabyung as depressive disorders, similar decreases were observed over the same period, at 144,579, 115,444 (−20.14%), and 112,914 (−21.89%), respectively (Table 1).

#### 3.1.3. Number of Claims (Yearly)

In 2012, 2016, and 2021, the annual number of claims for WM service by patients with depressive disorders was 3,976,933, 4,529,896 (+13.90%), and 7,124,984 (+79.16%), respectively, while the annual number of claims for the KM service was 20,788, 16,718 (−19.58%), and 12,907 (−37.91%), respectively (Figure 1c). When we considered stagnation syndrome and Hwabyung as depressive disorders, similar decreases were observed over the same period, at 141,875, 116,179 (−18.11%), and 113,556 (−19.96%), respectively (Table 1).

#### 3.1.4. Cost (Yearly)

In 2012, 2016, and 2021, the government insurance total cost (KRW) by WM service user in patients with depressive disorders 209,434,111, 258,367,214 (+23.36%), and 473,451,960 (+126.06%), respectively, while the government insurance total cost by KM service was 498,802, 456,074 (−8.57%), and 431,969 (−13.40%), respectively (Figure 1d). However, when we considered stagnation syndrome and Hwabyung as depressive disorders, the cost by KM service was increased to 2,954,019, 2,994,491 (+1.37%), and 4,587,991 (+55.31%), respectively (Table 1). A similar trend was also observed in the out-of-pocket total cost. In 2012, 2016, and 2021, the out-of-pocket total cost (KRW) by WM service user in patients with depressive disorders 142,322,713, 175,367,976 (+23.21%), and 362,431,453 (+154.65%), respectively, while the out-of-pocket total cost by KM service was 367,842, 334,814 (−8.98%), and 322,146 (−12.42%), respectively (Figure 1e). However, when we considered stagnation syndrome and Hwabyung as depressive disorders, the cost by KM service was increased to 2,188,161, 2,208,916 (+0.95%), and 3,312,030 (+51.36%), respectively (Table 1).

### 3.2. Efforts to Scientificize KM in the Context of Depressive Disorders

#### 3.2.1. Clinical Practice Guidelines

In South Korea, an evidence-based KM CPG for depression (1^st^ edition) was published in 2015 and developed in collaboration with KIOM and the Korean Society of Oriental Neuropsychiatry, consisting of 20 researchers in total [23]. They used a systematic review methodology and critically evaluated the collected evidence to provide recommendations, grades of recommendation, and level of evidence on the effectiveness and safety of herbal medicine, acupuncture, electro-acupuncture, qigong, meditation, and relaxation on individuals with depressive disorders [23]. From 2021, the second edition of this CPG is currently being developed by the Korean Society of Oriental Neuropsychiatry. It is stated that this CPG is scheduled to be released in February 2024. The clinical questions used to develop the second edition are summarized in Supplementary Table S1. It is stated that the effectiveness and safety of herbal medicine, acupuncture, and mind-body medicine on depressive disorders will be systematically analyzed.

#### 3.2.2. Research and Development

In South Korea, from 2007 to April 2022, a total of 17 kinds of R&D projects of KM for depression were started with funding from a government department. Most of these projects (12/17, 70.59%) were carried out by researchers affiliated with the university, followed by government-funded research institute (4/17, 23.53%), and small and medium business (1/17, 5.88%). The most common project topic was to elucidate the underlying therapeutic mechanism of herbal medicine or its components which can be used for depression (8/17, 47.06%). There were two projects related to KM CPG for depression, one to develop the second edition of evidence-based KM CPG for depression, and the other one to conduct randomized controlled clinical trials of herbal medicine (e.g., Banhahubak-tang for individuals with depression) for the development of the CPG. Interestingly, the project, launched in April 2022, seeks to synthesize the clinical evidence for KM treatment for suicidal behaviors. The research director stated that the purpose of this project was to establish the rationale for the participation of KM doctors in the suicide-crisis issue in South Korea, through the systematic review and analysis of healthcare big data.

## 4. Discussion

### 4.1. Findings of This Study

**Figure 2:**
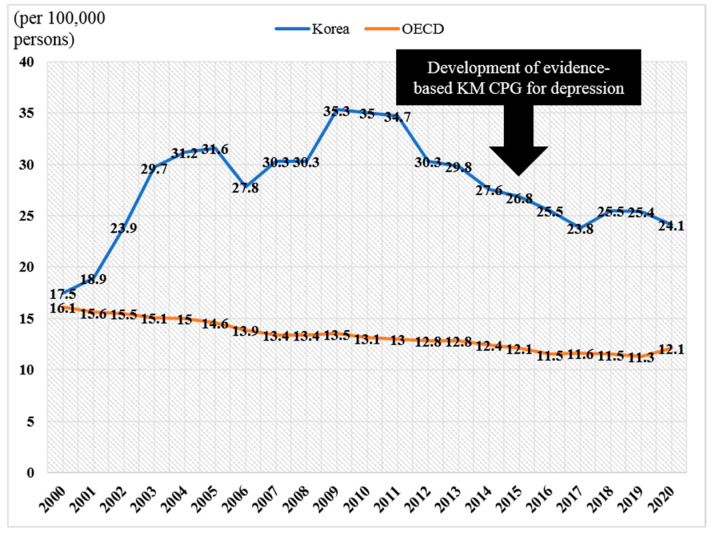
Suicide rates in South Korea between 2000 and 2010. Abbreviations. CPG, clinical practice guideline; OECD, Organization for Economic Co-operation and Development; KM, Korean medicine. Note. The data are derived from the OECD’s Health Status—Suicide Rates. (URL: https://data.oecd.org/healthstat/suicide-rates.htm accessed on 25 November 2022).

According to the findings, the use of KM treatment for patients with a depressive disorder appears to be gradually decreasing in South Korea, while the use of WM treatment is increasing. On the other hand, the government insurance total cost and out-of-pocket total cost related to the use of KM by patients with a depressive disorder in South Korea increased, which may reflect an increase in prices, and which was markedly lower than the increase in the costs related to WM use. The Korean government and KM society have made efforts to develop KM services scientifically, and depressive disorders have been regarded as one of its topics. In South Korea, evidence-based KM CPG for depression was developed and distributed in 2015 [23]. Moreover, efforts to revise this manual are currently ongoing with the support of the Korean government, and the revised version will be published in 2024. A number of R&D projects supporting KM for depression have also been conducted from 2007, and a project investigating the effect of KM treatment on suicidal behavior has recently been initiated. However, these efforts to scientificize KM for depression do not appear to have contributed to the reduction in suicide rates in Korea (Figure 2).

### 4.2. Some Challenges to Fill the Gap

Our findings highlight the gap between the accumulation of clinical evidence or the popularity of CIM for depressive disorders and its practical use in South Korea. The author would like to propose the following challenges to fill this gap. First, it is not yet clear why the use of KM for the treatment of patients with a depressive disorder is declining. However, there is still no study in South Korea that has investigated the perception of the use of KM service in patients with a depressive disorder in cross-section or longitudinal sections. Not only the perceptions of patients, but also the knowledge, perceptions, and attitudes of KM or WM doctors and policy makers are likely to contribute to changes in the use of KM services for depressive disorders in South Korea. Therefore, a survey of patients, clinicians, and policy makers, to investigate the knowledge and perceived needs of KM and other CIM modalities for patients with depressive disorders, may be needed. Second, the effectiveness and safety of CIM for depressive disorders in terms of EBM should be well-documented, including in the form of an evidence-based KM CPG. The evidence-based KM CPG for depression, currently being developed by the Korean Society of Oriental Neuropsychiatry, includes 42 clinical questions (Supplementary Table S1), and some questions are on the combination of KM with conventional treatments for depressive disorders (e.g., antidepressants and cognitive behavioral therapy). Recommendations based on these clinical questions may contribute to establishing a multidisciplinary management strategy using CIM in the context of national suicide-prevention strategies in South Korea. The third challenge is academic communication between WM and KM doctors to treat depressive disorders in South Korea. In other words, in the context of a high suicide rate, efforts to develop an integrative and multidisciplinary model using both WM and KM for depressive disorders may be needed. Importantly, the long-lasting high suicide rate in South Korea demonstrates the necessity of providing better mental health services than currently exist. Considering that suicide-prevention strategies require a multidisciplinary approach [9], and that KM is an underutilized for patients with depressive disorders in South Korea, developing an integrative model incorporating KM and WM to improve depressive disorders in South Korea may provide one solution to the suicide issue in South Korea. Fourth, if KM is used to build a multidisciplinary strategy for suicide prevention in South Korea, culturally relevant multidisciplinary strategy construction could be possible. Previous studies have emphasized the need for culturally appropriate interventions for suicide prevention in an intercultural context [24,25,26]. KM and some CIM modalities (e.g., herbal medicine and acupuncture), which are particularly familiar to East Asians, can be of great selective benefit to populations who perceive this treatment as favorable and familiar. More importantly, in Asian countries, depressive disorders are more likely to be expressed as somatic symptoms (i.e., somatization) [27], and patients with medically unexplained somatic symptoms tend to prefer CIM [28]. Patients who received acupuncture with medically unexplained somatic symptoms were reported to perceive a range of positive effects, including improvements in mental health [29,30]. Therefore, acupuncture or acupuncture procedures [31] may provide a beneficial effect physically and psychologically to patients with a depressive disorder who are ‘hidden’ in their somatic symptoms, and it has the potential to contribute to managing the risk of suicide in patients with a depressive disorder that has not been met by conventional medicine. However, it should be further elucidated whether CIM treatment, including acupuncture, is helpful not only to relieve depression but also to prevent suicide. Encouragingly, a recent systematic review protocol reported a plan to investigate the effects of acupuncture on suicidal behavior [32], and the results will be helpful for understanding the effects of acupuncture on suicidal behavior, not limited to the context of depressive disorders.

### 4.3. Limitations of This Study

One of the limitations of this study was that individual KM treatments (e.g., acupuncture, herbal medicine, cupping) were not analyzed. Therefore, this study did not analyze changes in the use of individual KM treatment in Koreans with a depressive disorder. Furthermore, since CIM can be performed at non-medical institutions other than KM clinics in South Korea, this analysis cannot confirm the overall CIM use or needs of depressive-disorder patients in South Korea. For the same reason, changes in the use of individual WM treatments for depressive disorders were not analyzed. Secondly, there is a possibility that patients with depressive disorders were treated for other conditions, especially somatic symptoms, at the KM clinic. This may be related to the tendency of Asian patients with a depressive disorder to have more somatic symptoms [27]. However, such a tendency does not fully explain the steady decrease in the rate of use of KM services by patients with depressive disorders in South Korea. The last limitation is that the data source used in this study includes only health insurance claim data. That is, in the analysis derived from this database, the medical cost for non-insured treatment was omitted. Since herbal decoction, which accounts for an important portion of KM treatment, is not covered by health insurance in South Korea, the cost results related to KM services in this analysis are potentially underestimated.

## 5. Conclusions

In this report, the author reports a declining use of KM for the treatment of depressive disorders in South Korea, which has the highest suicide rate among OECD countries. This is contrary to the Korean government’s efforts to promote the evidence-based use of KM for depressive disorders. Studies examining the challenges to KM use in depressive disorders may provide clues to solving the problem of mental disorders and higher suicide rates in South Korea in the future.

## Figures and Tables

**Table 1:** The use of Western and Korean medicine in patients with depressive disorders over the past 10 years in South Korea (2012–2021).

Year	2012	2013	2014	2015	2016	2017	2018	2019	2020	2021
Number of patients (yearly)										
WM	662,947	655,623	646,763	660,534	704,799	743,010	826,733	873,380	914,087	989,909
KM model 1	3607	3203	3233	2881	2955	2686	2398	2381	2195	2151
KM model 2	31,539	29,135	28,081	25,784	24,686	23,973	24,119	23,965	21,473	21,072
Number of visit days (yearly)										
WM	4,639,309	4,735,159	4,720,017	4,814,426	5,176,296	5,418,846	6,048,370	6,473,779	6,816,545	7,555,597
KM model 1	22,039	18,707	17,633	16,859	17,082	16,076	13,704	13,977	13,194	12,812
KM model 2	144,549	132,134	129,542	120,657	115,444	113,517	119,264	118,280	112,400	112,914
Number of claims (yearly)										
WM	3976,933	4,094,317	4,076,407	4,187,329	4,529,896	4,829,436	5,449,632	5,910,574	6,347,931	7,124,984
KM model 1	20,788	18,100	17,310	16,327	16,718	15,576	13,183	13,692	13,084	12,907
KM model 2	141,875	131,532	129,307	120,413	116,179	114,383	119,877	118,762	112,993	113,556
Government insurance total cost (yearly, KRW)										
WM	209,434,111	217,667,489	223,455,299	231,976,757	258,367,214	276,993,933	331,941,392	377,699,064	408,073,951	473,451,960
KM model 1	498,802	446,376	442,770	447,315	456,074	433,248	414,205	442,717	438,357	431,969
KM model 2	2,954,019	2,707,927	2,842,348	2,759,091	2,994,491	3,353,658	3,974,904	4,252,291	4,482,472	4,587,991
Out-of-pocket total cost (yearly, KRW)										
WM	142,322,713	147,709,895	152,413,421	157,953,108	175,367,976	187,632,629	234,485,129	283,271,858	310,507,839	362,431,453
KM model 1	367,842	328,453	325,846	329,277	334,819	319,792	308,718	330,653	327,093	322,146
KM model 2	2,188,161	2,010,743	2,117,269	2,049,623	2,208,916	2,466,432	2,936,312	3,111,706	3,248,129	3,312,030

Abbreviations. KM, Korean medicine; WM, Western medicine. Note. In KM model 1, patients with depressive disorders were defined as having a main diagnosis of F32, F320, F321, F322, F323, F328, F329, F33, F330, F331, F332, F333, F334, F338, or F339 (International Classification of Diseases code). In KM model 2, in addition to the depressive disorders defined in model 1, U221 and U222 (International Classification of Diseases code), which are considered mental disorders similar to depression in KM, were also considered depressive disorders. As of July 2022, 1000 KRW was equivalent to 0.75 USD.

## Data Availability

The data extracted from the studies included and data used for all analyses were all included in this manuscript.

## Supplementary Materials

The following supporting information can be downloaded at: https://www.mdpi.com/article/10.3390/jcm11237022/s1, Table S1: Clinical questions of Korean medicine clinical practice guideline for depressive disorders, being developed by the Korean Society of Oriental Neuropsychiatry (2021–2024).
