# Supplementary material for: Gap between the Scientificization and Utilization of Korean Medicine for Depressive Disorder in South Korea with the Highest Suicide Rate among OECD Countries
Source: J Clin Med. 2022 Nov 28;11(23):7022. doi: 10.3390/jcm11237022 (PMC9737407; doi:10.3390/jcm11237022)
Supplement: Supplementary file 1 [file jcm-11-07022-s001.zip › jcm-2032009-supplementary.pdf]

Table S1. Clinical questions of Korean medicine clinical practice guideline for depressive disorders, being developed by the Korean Society of Oriental Neuropsychiatry (2021-2024).

Intervention	Clinical questions
**Herbal medicine as monotherapy**	Q1. Does herbal medicine treatment improve depression scale scores compared to antidepressants in adults with major depressive disorder?; Q2. Does herbal medicine treatment improve depression scale scores compared to antidepressants in patients with postpartum depression?; Q3. Does herbal medicine treatment improve depression scale scores compared to antidepressants in patients with menopausal depression?; Q4. Does herbal medicine treatment improve depression scale scores compared to antidepressants in elderly with major depressive disorder?; Q5. Does herbal medicine treatment improve depression scale scores compared to antidepressants in cancer patients with depression?
**Herbal medicine as combined therapy**	Q6. Does the combination treatment of herbal medicine and antidepressants improve depression scale scores compared to antidepressants alone in adults with major depressive disorder?; Q7. Does the combination treatment of herbal medicine and acupuncture improve depression scale scores compared to antidepressants in adults with major depressive disorder?; Q8. Does the combination treatment of herbal medicine and acupuncture improve depression scale scores compared to herbal medicine alone in adults with major depressive disorder?; Q9. Does the combination treatment of herbal medicine and acupuncture improve depression scale scores compared to antidepressants alone in patients with postpartum depression?; Q10. Does the combination treatment of herbal medicine and antidepressants improve depression scale scores compared to antidepressants alone in patients with menopausal depression?; Q11. Does the combination treatment of herbal medicine and antidepressants improve depression scale scores compared to antidepressants alone in elderly with depression?; Q12. Does the combination treatment of herbal medicine and antidepressants improve depression scale scores compared to antidepressants alone in cancer patients with depression?; Q13. Does the combination treatment of herbal medicine and psychotherapy improve depression scale scores compared to

	psychotherapy alone in cancer patients with depression?
**Other issues on herbal medicine**	Q14. When treating adults with major depressive disorder, is it more effective to treat according to the pattern (Zheng)?; Q15. What is the appropriate period of taking herbal medicine to treat adults with major depressive disorder?
**Acupuncture as monotherapy**	Q1. Does acupuncture improve depression scale scores compared to antidepressants in adults with major depressive disorder?; Q2. Does acupuncture improve depression and pain compared to conventional treatment (i.e., antidepressants) in depressed patients with chronic pain condition?; Q3. Does acupuncture improve depression scale scores compared to antidepressants in cancer patients with depression?; Q4. Does acupuncture administered to depressed patients during pregnancy improve depression scale scores and reduce the incidence of postpartum depression compared to sham acupuncture?; Q5. Does acupuncture improve depression scale scores compared to sham acupuncture in patients with postpartum depression?; Q6. Does acupuncture improve depression scale scores compared to antidepressants in patients with postpartum depression?; Q7. Does acupuncture improve depression scale scores compared to antidepressants in patients with menopause depression?; Q8. Does electro-acupuncture improve depression scale scores compared to antidepressants in adults with major depressive disorder?; Q9. Does electro-acupuncture improve depression scale scores compared to sham acupuncture in patients with postpartum depression?; Q10. Does electro-acupuncture improve depression scale scores compared to antidepressants in patients with menopause depression?
**Acupuncture as combined therapy**	Q11. Does the combination treatment of acupuncture and conventional treatment (i.e., antidepressants) improve depression scale scores compared to combination treatment of counselling and conventional treatment or conventional treatment alone in adults with major depressive disorder?; Q12. Does the combination treatment of acupuncture and antidepressants improve depression scale scores compared to antidepressants alone in adults with major depressive disorder?; Q13. Does the combination treatment of electro-acupuncture and antidepressants improve depression scale scores compared to antidepressants alone in adults with major depressive disorder?; Q14. Does the

	combination treatment of acupuncture and moxibustion improve depression scale scores compared to antidepressants alone in adults with major depressive disorder?
**Other issues on acupuncture**	Q15. What type of acupoints should be preferred for acupuncture treatment for patients with depression?
**Mind-body medicine**	Q1. Does qigong improve depression scale scores compared to wait-list in adults with major depressive disorder?; Q2. Does qigong improve depression scale scores compared to walking in adults with major depressive disorder?; Q3. Does qigong improve depression scale scores compared to control (wait-list or walking) in patients with menopause depression?; Q4. Does taichi improve depression scale scores compared to control (education program) in adults with major depressive disorder?; Q5. Does the combination treatment of meditation and antidepressants improve depression scale scores compared to antidepressants alone in patients with major depressive disorder (treatment-resistant)?; Q6. Does the combination treatment of meditation and conventional treatment improve depression and quality of life, and lower the recurrence of depressive episode compared to conventional treatment alone in patients with depression in remission?; Q7. Does the combination treatment of meditation and antidepressants improve depression and quality of life, and lower the recurrence of depressive episode compared to antidepressants alone in patients with depression in remission?; Q8. Does meditation improve depression scale scores compared to conventional treatment in elderly with moderate major depressive disorder?; Q9. Does the combination of online meditation and antidepressants improve depression scale scores compared to antidepressants alone in patients in their 20s with major depressive disorder?; Q10. Does relaxation improve depression scale scores compared to cognitive behavior therapy in adults with major depressive disorder?; Q11. Does relaxation improve depression scale scores compared to antidepressants in adults with major depressive disorder?; Q12. Does EEG-based neurofeedback improve depression scale scores compared to antidepressants in adults with major depressive disorder?
